# Non-invasive, transient determination of the core temperature of a heat-generating solid body

**DOI:** 10.1038/srep35886

**Published:** 2016-11-02

**Authors:** Dean Anthony, Daipayan Sarkar, Ankur Jain

**Affiliations:** 1 Mechanical and Aerospace Engineering Department, University of Texas at Arlington, Arlington, TX, USA

## Abstract

While temperature on the surface of a heat-generating solid body can be easily measured using a variety of methods, very few techniques exist for non-invasively measuring the temperature inside the solid body as a function of time. Measurement of internal temperature is very desirable since measurement of just the surface temperature gives no indication of temperature inside the body, and system performance and safety is governed primarily by the highest temperature, encountered usually at the core of the body. This paper presents a technique to non-invasively determine the internal temperature based on the theoretical relationship between the core temperature and surface temperature distribution on the outside of a heat-generating solid body as functions of time. Experiments using infrared thermography of the outside surface of a thermal test cell in a variety of heating and cooling conditions demonstrate good agreement of the predicted core temperature as a function of time with actual core temperature measurement using an embedded thermocouple. This paper demonstrates a capability to thermally probe inside solid bodies in a non-invasive fashion. This directly benefits the accurate performance prediction and control of a variety of engineering systems where the time-varying core temperature plays a key role.

While several experimental methods exist for measurement of temperature on the surface of a solid body^1^,^2^,^3^,^4^,^5^,^6^,^7^, there is a lack of experimental techniques non-invasive measurement of temperature inside the body. Development of a technique with similar capability for non-invasive internal temperature measurement will have a dramatic impact on performance, safety and reliability of engineering systems, each of which are directly affected by the peak temperature, which usually occurs at the core of the cell. Performance optimization of such systems, including Li-ion cells, is often carried out using surface temperature measurement, which may result in significant error, since surface temperature measurement gives very little indication of the core temperature, which may be much higher for heat-generating bodies.

While the core temperature may in principle be measured by inserting or embedding a temperature sensor inside the solid body^8^,^9^,^10^, and optimizing the location of the sensor and accompanying wires^11^, this approach is often too intrusive and impractical. As an example, the temperature at the core of a Li-ion cell is of much interest for performance optimization and safety^9^, however, due to the presence of electrochemical materials inside the hermetically sealed cell, insertion of a temperature sensor is simply not possible on a large scale. The use of the measured surface temperature for performance optimization and design of safety features is inappropriate, since the greater temperature at the core will govern the design of thermal management to ensure safety. For example, a thermal management system for heat removal based on surface temperature measurement alone is likely to fail due to much higher temperature inside the cell. Further, the advent of a safety problem such as thermal runaway can be accurately predicted by measuring the core temperature, and not the surface temperature^12^.

Further, it is also desirable that such a technique should be a direct thermal technique that does not depend on the conversion of temperature rise to another measurable physical parameter. For example, ultrasonic methods that utilize the temperature dependence of ultrasonic wave propagation in solids have been used in the past for estimating temperature profile within a solid body^13^,^14^. However, these methods only work on homogeneous bodies, and further, may not be rapid enough to provide transient temperature distribution.

A method for determining the steady-state core temperature of a solid body was recently developed^15^ based on measurement of the surface temperature distribution of the solid body in steady state. However, this technique was capable of determining only the steady-state core temperature, whereas the transient variation of the core temperature is often much more critical for several reasons. In many cases, the reliability and lifetime of an engineering system is governed by performance during transient operation, and not by steady state performance. Several engineering processes are inherently transient and do not reach a steady-state, thereby limiting the applicability of a steady-state core temperature measurement method. For example, a process for aggressively discharging a 26650 Li-ion cell may complete within 6–15 minutes^9^, whereas the thermal time constant for the cell is much longer^16^. In such a case, the capability of determining the transient core temperature in a non-intrusive fashion will help make real-time decisions to improve performance, safety and reliability. Finally, evolution of temperature of the body in time affects other physical parameters and processes, such as stresses, fatigue, etc.^17^. For these reasons, it is very desirable to develop a method to determine the core temperature of a heat-generating solid body as a function of time in a contactless, non-intrusive fashion.

This paper addresses this critical research need by developing a technique for non-intrusively determining the transient temperature inside a solid, heat-generating body. This method utilizes information from the outside surface temperature distribution measured as a function of time. Based on a general solution for thermal conduction in such a case, it is shown that there exists a relationship between the core temperature and outside surface temperature distribution as functions of time, which could be used to determine the core temperature without the need for physically accessing the core by using appropriate space and time integrals of the transient temperature distribution on the surface of the body. Surface temperature measurement is carried out using infrared thermography on a thermal test cell capable of internal heat generation, and the core temperature is predicted as a function of time using these data. A thermocouple embedded at the core of this cell provides the actual core temperature measurement, which is used to validate the technique. Core temperature determined through this technique is found to be in close agreement with the actual temperature over the entire experimental period in a variety of heating and cooling conditions.

## Results and Discussion

### Theoretical expression for transient core temperature as a function of surface temperature distribution

Consider a heat-generating infinite cylinder of radius R with constant, volumetric heat generation *Q* within the cylinder, shown schematically in Supplementary Figure S1. Assume the radial thermal conductivity and heat capacity of the cylinder to be *k*_*r*_ and *C*_*p*_ respectively. The surface temperature distribution around the periphery of the cylinder, *T*_*0*_(*θ,t*) is assumed to be known through a measurement. This is a direct thermal conduction problem, variants of which have been solved in the past^18^. For example, a solution for thermal conduction in a heat-generating, isotropic cylinder with a circumferentially uniform temperature imposed on the outer surface has been presented using the method of Green’s functions^18^. In this work, a solution is derived to account for circumferential variation in the outside temperature, as well as thermal conductivity orthotropy within the cylinder, both of which are relevant to realistic engineering systems. The detailed derivation in Supplementary Information shows that the temperature at the core of the cylinder, *r *=* 0*, is given as a function of time by


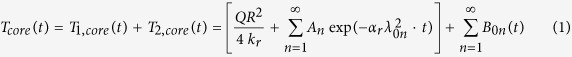


In equation (1),


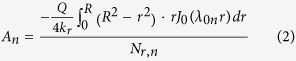


and,


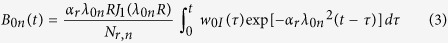


Further, in equations (2) and (3), *J*_*m*_ refers to the Bessel function of the first kind and order *m*, and 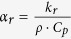
is the radial thermal diffusivity. Eigenvalues *λ*_*0n*_ are obtained from the roots of *J*_0_, and the radial norm *N*_*r,n*_ is given by


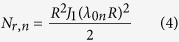


In equation (3), *w*_*0I*_(*τ*) is the circumferentially averaged value of the measured surface temperature *T*_*0*_(*θ,t*), given by


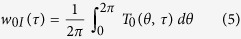


In case the heat generation rate within the cell changes with time, given by *Q*(*t*), the relationship between the core temperature and surface temperature distribution is given by


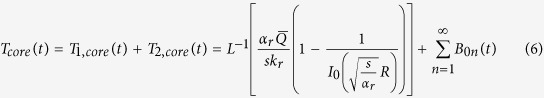


Where *L*^−*1*^ refers to the inverse Laplace transform, 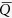 is the Laplace transform of *Q*(*t*), and *I*_*0*_ is the modified Bessel function of the first kind and of order zero.

Detailed derivation of equations (1) and (6) is discussed in Supplementary Information.

The solution derived here is along similar lines as one presented by Özişik^18^, except that the present solution additionally accounts for circumferential variation of the outside temperature, as well as orthotropy in thermal conductivity within the cylinder. Both of these realistic effects are important to model, since from convective heat transfer theory, temperature on the cylinder surface is known to vary circumferentially, as also evidenced by experimental data described later. Further, in the case of Li-ion batteries, a strong thermal conduction orthotropy is known to exist^19^.

While Özişik’s solution used the Green’s function approach, this paper uses Fourier series expansion and Laplace transforms for time-varying heat generation in order to be consistent with the constant heat generation case. Since heat generation rate for a Li-ion cell is usually available at discrete time steps through experimental measurements^20^ or numerical simulation of electrochemistry^21^, both of these approaches may need numerical integration – for evaluation of time-domain integral of *Q*(*t*) in Green’s function approach, and for evaluation of inverse Laplace transforms in the Laplace transform approach.

Supplementary Figure S2 compares the transient core temperature based on this theoretical model with finite-element simulations of the transient temperature field. Refinement in mesh and timestep discretization is carried out in the finite-element simulations until no further change in the solution occurs upon further refinement. The simulation uses approximately 528 K elements and 100 timesteps through the entire duration. Two cases are considered with constant and time-varying outside surface temperature. In each case, there is excellent agreement between the theoretical model and finite-element simulation results, with a peak difference between the two of 0.47 °C and 0.70 °C respectively.

Equations (1) and (6) show that the core temperature at any time can be determined through an integral involving the circumferentially-integrated surface temperature distribution at all times prior to the time of interest. This relationship is the basis for a technique for determining the core temperature of a cylinder without invasively inserting a sensor within, but instead by measuring the temperature distribution on its outer surface as a function of time, which could be done using a variety of methods such as infrared thermography. Detailed derivation in Supplementary Information shows that in a thermally orthotropic cylinder, while the general temperature field in the cylinder is a function of the circumferential thermal conductivity *k*_*θ,*_determining the core temperature requires information about the radial thermal conductivity *k*_*r*_, but not *k*_*θ*_.

### Temperature measurement results

**Figure 1:**
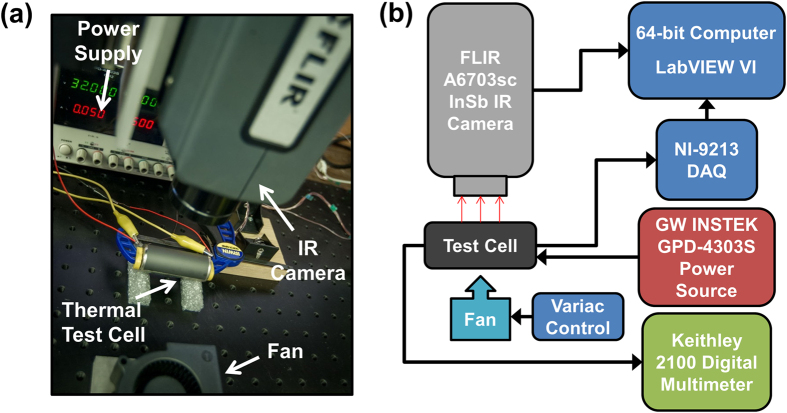
(**a**) Picture of the experimental setup for non-invasive transient core temperature measurement, (**b**) Schematic of the experimental setup and measurement method.

**Figure 2:**
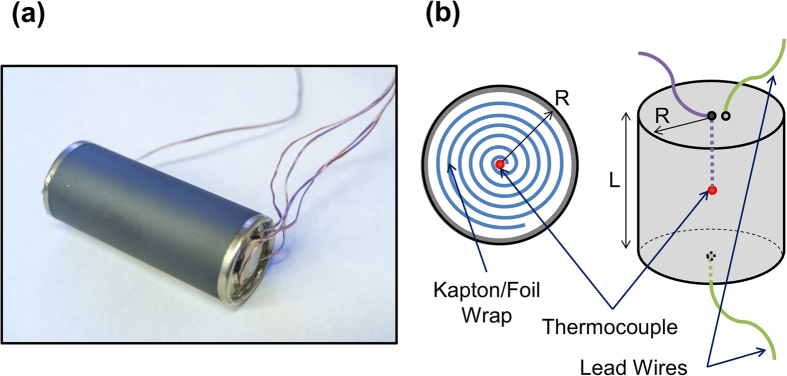
Schematic of the thermal test cell (13 mm radius and 65 mm height) with internal volumetric heat generation and embedded core thermocouple.

Experiments are carried out to non-invasively determine the transient evolution of core temperature of a cylindrical thermal test cell using infrared measurements of the surface temperature distribution based on the model presented in the previous sub-section. The thermal test cell comprises a tightly wound roll of resistive metal foil assembled inside a casing with a thermocouple embedded at the core of the roll. Electric current passing through the metal foil is used to generate heat at a desired rate throughout the cell volume through Joule heating. Measurements from the embedded thermocouple provide the actual core temperature, which can be used to validate the technique to determine the core temperature using the surface temperature measurement and equations (1) and (6). A number of heat generation rates and external cooling conditions are investigated. Radial thermal conductivity *k*_*r*_ is obtained from a past measurement^15^ on the same thermal test cell using an adiabatic heating method where the thermal response of the cell to external heating is used to determine its thermal properties^19^. Heat capacity *C*_*p*_ is determined from the mass-weighted average of heat capacities of individual material components of the thermal test cell. Figure 1(a,b) show a picture and schematic of the experimental setup. Figure 2(a,b) show a picture and a schematic of the thermal test cell, showing the volumetric heat generation in the metal foil, as well as the location of the embedded thermocouple.

Supplementary Figure S3 plots the surface temperature distribution as a function of *θ* measured by the infrared camera at a number of times following the start of 0.86 W heating within the test cell in free convection cooling conditions. The temperature distribution evolves with time, although the nature of *θ*-dependence remains nearly invariant. There is sharp increase in temperature initially, whereas the rate of change in temperature reduces, as expected, as the test cell approaches thermal steady state.

**Figure 3:**
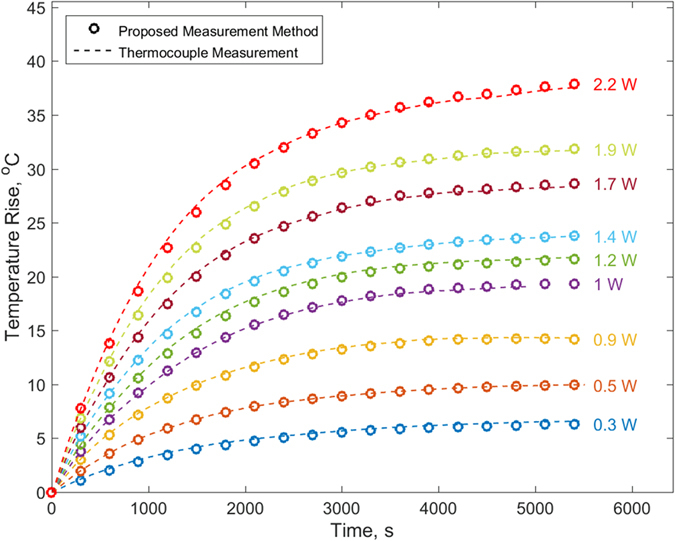
Predicted core temperature as a function of time for a number of heating powers in free convection cooling conditions. Actual temperature measured by embedded thermocouple is also shown as broken lines for each power.

Figure 3 plots the calculated core temperature for transient experiments at a number of heating powers in free convection cooling conditions. In brief, the surface temperature measurements *T*_*0*_(*θ,τ*) shown in Supplementary Figure S3 are integrated at each time *τ* to determine *w*_*0I*_(*τ*) according to equation (5). Coefficients A_n_ and *B*_*0n*_(*t*) are then determined using equations (2) and (3) respectively. Note that computing the latter coefficient at any given time requires information about *w*_*0I*_ at that time and at all prior times. Finally, the core temperature is determined as a function of time using equation (1). For comparison, the core temperature measured by the embedded thermocouple in the test cell for each power is also plotted in Figure 3. There is excellent agreement over the entire experiment duration between the core temperature predicted by the technique and actual core temperature measurement from the embedded thermocouple. This agreement holds for multiple heating powers. The residual, defined as the difference between measured and analytical temperature rise during an experiment is 0.76 °C at most, which is well within the experimental measurement uncertainty discussed later. In general, the mean of these residuals over the experimental period increases as power increases, but even for measurements at the highest power of 2.2 W when the temperature rise is around 37 °C, the mean and standard deviation of the residuals is only 0.30 and 0.21 °C respectively. Supplementary Figure S4 plots the maximum residual between measured and analytical temperature rise as a function of the heating power. In each case, the maximum residual is lower than the experimental uncertainty.

**Figure 4:**
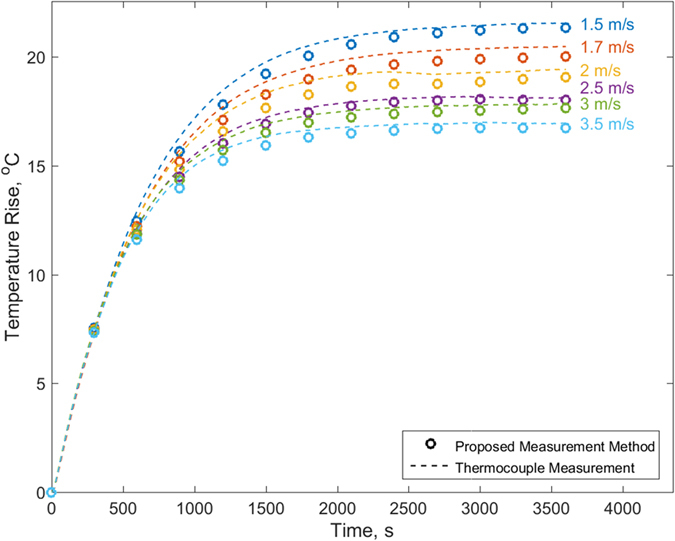
Comparison of measured core temperature as a function of time with measurement from core-embedded thermocouple for 2.2 W heating power with different air speeds from a cooling fan.

To further investigate the performance of this technique, measurements are repeated in the presence of forced convection cooling due to air flow from a fan. Figure 4 plots the core temperature determined as a function of time for a heating power of 2.2 W and at different air speeds. As expected, the temperature reduces as air speed increases. At each air speed, there is excellent agreement between the technique discussed in this paper, and thermocouple measurements of the core temperature. The peak residual between the two over the entire experimental duration is 0.75 °C. The residuals are higher at larger times, but even in the worst case, much below the resolution of the measurement technique. The highest mean and standard deviation of these residuals is 0.46 and 0.23 °C when the temperature rise itself is around 18 °C. These numbers are similar to statistics for the previous Figure.

Time-varying heat generation is often encountered in energy conversion systems. To evaluate the accuracy of this technique in such conditions, a set of experiments are carried out with a time-varying heat generation rate in the thermal test cell. *Q*(*t*) profiles representative of high-rate cyclic discharge of a Li-ion cell^9^ are used. In this case, heat is generated at a large rate during the discharge process, whereas the heat generation rate is much lower or zero during the charge and rest periods between successive discharges. In these experiments, Joule heating in the test cell is switched on and off at 15 minute intervals for three cycles. Experiments are carried out at different powers and in different cooling conditions. Experimental measurements of the surface temperature distribution are analyzed using the theoretical model to determine the core temperature as a function of time. The inverse Laplace transformation in equation (6) is carried out using de Hoog’s quotient difference method^22^ as implemented by Hollenbeck^23^.

**Figure 5:**
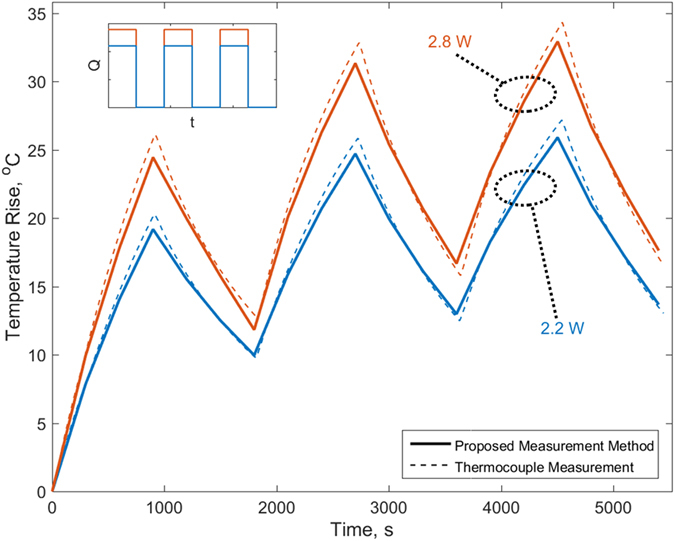
Comparison of measured core temperature as a function of time with measurement from core-embedded thermocouple for three cycles of ON-OFF heating with two different heating powers in identical cooling conditions.

Figure 5 plots the core temperature as a function of time predicted by this technique for two different heating powers with free convection cooling. The actual core temperature measurement from the embedded thermocouple is also plotted for both cases. Figure 5 shows that the core temperature is predicted accurately during both parts of the cycles, and over multiple cycles. The peak temperature reached in each cycle is under-predicted only by a few percent.

**Figure 6:**
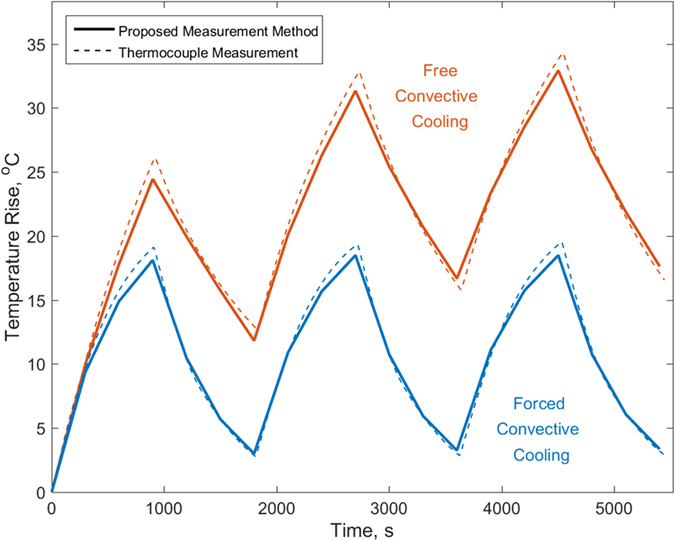
Comparison of measured core temperature as a function of time with measurement from core-embedded thermocouple for three cycles of ON-OFF heating with 2.78 W heating power in natural convection and forced convection cooling conditions.

Figure 6 compares the core temperature with embedded thermocouple data for two cases where the heating power is held constant at 2.78 W, with two different cooling conditions – free convection in the absence of air flow, and forced convection with a 2.5 m/s air flow. As expected, the core temperature reduces when air flow is introduced, and in each case, the core temperature determined from this technique is in good agreement with thermocouple data.

The experiments carried out here, as summarized in Figures 3 through 6 demonstrate the capability of this technique to accurately determine the core temperature of the cell in a wide variety of experimental conditions including heating powers, cooling conditions and time-varying heating. In each case, the core temperature is determined as a function of time with very good accuracy in a non-intrusive fashion using only surface temperature data. While this paper discusses this technique in the context of a cylindrical body, the technique can be extended easily to bodies of other shapes, as long as an analytical derivation similar to the derivation shown in Supplementary Information can be carried out to determine a relationship between the core temperature and surface temperature of the body.

### Convergence of the theoretical model

Supplementary Figure S5 plots the transient core temperature calculation from equation (1) for different numbers of eigenvalues for the *B*_*0n*_(*t*) term for 0.86 W heating case with free convection cooling. Measurement from the embedded thermocouple is also plotted for comparison. This plot shows that at least 400 eigenvalues are needed for good agreement between the model and thermocouple data. Several other analytical thermal models developed in the past for other applications have required far fewer number of eigenvalues^24^,^25^. However, the large number encountered here is not unexpected. In a limiting case where a constant outside surface temperature *T*_*out*_ is imposed on the outer surface of the cylinder with no internal heat generation, it can be shown using equation (1) that the core temperature at large times is given by


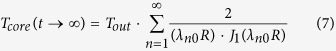


The infinite series in equation (7) can be shown to converge to a value of 1, as expected, but, as shown in Supplementary Figure S6, this convergence is very slow, requiring at least 400 eigenvalues for reaching within 4% of the infinite series sum. This, along with the fact that the outside surface temperature itself changes with time in actual experiments explains the reason for requiring a large number of eigenvalues for accurately computing the model.

### Calibration and validation of infrared-based surface temperature measurements

The accuracy of the technique to determine the core temperature depends critically on the accuracy of surface temperature measurement, carried out in this case with an infrared camera. Experiments are carried out to establish the accuracy of the infrared temperature measurements. In these experiments, the infrared camera is used to measure the temperature of a thermal stage as a function of time. The temperature of the graphite-coated stage is well known through a thermocouple embedded in the stage. Supplementary Figure S7 plots the temperature of the stage during a ramp up from room temperature to 60 °C at a rate of 5 °C/min. The plot compares measurement from the infrared camera with the actual stage temperature measured by the embedded thermocouple. These data clearly show that the infrared camera, when calibrated for the graphite surface is able to accurately measure the surface temperature as a function of time. The peak deviation between the two measurements is 0.31 °C, which is well within the measurement uncertainty of the infrared camera and the embedded thermocouple. Since the rate of temperature change in core temperature experiments is lower than the ramp rate chosen for these validation experiments, these data establish the accuracy of infrared based transient surface temperature measurement.

### Sensitivity and uncertainty analysis

An uncertainty analysis is carried out to determine the overall uncertainty in determining the core temperature, as well as to identify key sources of uncertainty. Experimental uncertainty in the core temperature arises primarily from uncertainties in measurement of heat generation *Q*, radial thermal conductivity *k*_*r*_, and surface temperature field *T*_*0*_(*θ*,*t*) Uncertainty in heat generation rate is expected to be very small since it is measured electrically using precise measurement instruments. Based on error propagation analysis^26^ in experimental measurement of thermal conductivity *k*_*r*_ carried out in the recent past^15^,^19^, the uncertainty in thermal conductivity measurement has been estimated to be ±5%^19^. This represents the expected uncertainty in the thermal conductivity measurement due to propagation of errors involved in measurement of quantities needed for determining thermal conductivity. The uncertainty in infrared-based surface temperature measurement is obtained from the calibration curve (Supplementary Figure S7) to be 0.31 °C. An error propagation analysis^26^ of equation (1) shows that the overall uncertainty in core temperature measurement is expected to be around ±10%.

**Figure 7:**
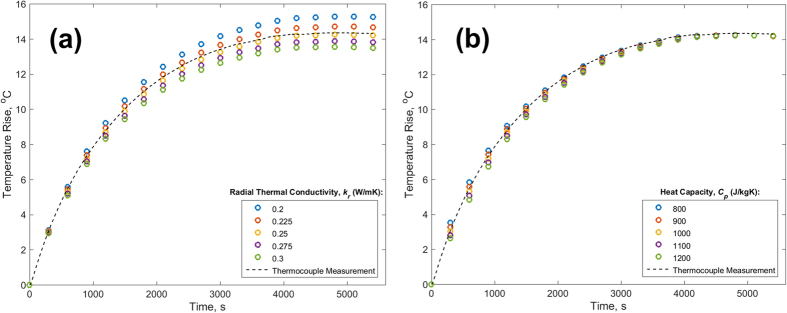
Sensitivity of the measured transient core temperature profile to (**a**) radial thermal conductivity, and (**b**) heat capacity for 0.86 W heated cylinder in free convective cooling conditions. Core temperature as a function of time from the embedded thermocouple is also plotted as a broken line.

Radial thermal conductivity *k*_*r*_ and heat capacity *C*_*p*_ of the cylinder influence the predicted core temperature as a function of time for the infinite cylinder. Note that the circumferential thermal conductivity *k*_*θ*_ is not required. The sensitivity of measurement results to these thermal properties is examined in Figure 7(a,b), that plot the predicted core temperature as a function of time for different values of *k*_*r*_ and *C*_*p*_ respectively for a specific case of 0.86 W heating in natural convection conditions. The core temperature measured by the thermocouple is also plotted for reference. Figure 7(a) shows that the core temperature is somewhat sensitive to the value of *k*_*r*_, indicating that it is important to accurately know beforehand the radial thermal conductivity of the cylinder. For measurements discussed in this paper, *k*_*r*_ is measured using a recently developed anisotropic thermal conductivity measurement method^19^, and the deviation of core temperature measurements from thermocouple data is within the uncertainty associated with *k*_*r*_ measurement and other sources of uncertainty. Figure 7(b) presents a similar plot for the variation of core temperature for different values of *C*_*p*_, indicating that unlike *k*_*r*_, the results are not very sensitive to the value of heat capacity.

The key limitation of the non-invasive core measurement method discussed here is that information about the thermal conductivity, heat capacity and heat generation rate of the body must be known in advance. While the thermal properties may be known if the body is made of a standard material, or through separate thermal property measurements, it may present challenges if the body is a composite material. In such a case, the use of effective thermal conductivity and heat capacity may be appropriate. Information about the heat generation rate will typically come from the mechanism of heat generation. For example, for Joule heating, the heat generation rate may be obtained from current and potential difference measurements. For heating due to chemical reactions, heat generation rate may be obtained from the enthalpies and rates of reactions.

## Methods

### Theoretical modeling

A derivation for the relationship between the core temperature as a function of time, *T*_*core*_(*t*) and the surface temperature distribution on the outside surface, *T*_*0*_(*θ,t*) for an infinite cylinder is carried out using the method of undetermined parameters for solving the governing energy equation with boundary conditions that capture the measured outside surface temperature distribution. In addition, the method of Laplace transforms is used for the case of time-varying heat generation rate. Detailed derivations are provided as Supplementary Information.

### Fabrication of thermal test cell

Experiments described in this paper utilize a thermal test cell of 13 mm radius and 65 mm height that is capable of uniform volumetric heat generation through Joule heating in metal foil rolled and embedded within the test cell, as shown in Figure 2(a,b). Joule heating provides the advantage of close control of heat generation rate through the input electric current, and of varying heat generation as a function of time. Fabrication of the test cell has been described in detail previously^15^. In brief, a thin metal foil, insulated with Kapton tape is rolled around a thin rod and inserted inside a metal casing. A T-type thermocouple is inserted at the core of the roll at mid-height. Electric current can be passed through the metal foil through two thin metal wires. Remaining space within the casing is filled with a temperature-curable polymer, poly-dimethylsiloxane (PDMS), following which, the test cell is sealed. Air bubbles in the uncured PDMS monomer are removed in a vacuum desiccator. The curing process is allowed to occur at room temperature over 24 hours to further facilitate removal of bubbles.

The thermal test cell is designed to have similar geometry and thermal properties as a 26650 Li-ion cell, which is commonly used for high density electrochemical energy storage and conversion^27^. Heat capacity of the test cell is determined through a weighted average of the well-known heat capacities of all individual components including metal foil, Kapton tape, PDMS, metal casing, etc. Radial thermal conductivity of the cell is measured through a recently developed adiabatic heating method^19^. In brief, a thermocouple is placed at the outer surface of the test cell at mid-height using a thermal epoxy. A flexible heater is then wrapped around the outer curved surface of the test cell. The test cell is then wrapped in insulation tape, and placed inside a vacuum chamber. Transient temperature rise of the cell measured by the thermocouple, in response to a DC heat flux, is compared with an analytical thermal model to determine the radial thermal conductivity of the cell. Specifically, the intercept of the temperature vs. time plot is used to determine the radial thermal conductivity of the cell. Details of this method have been presented recently^19^. Note that the other two components of the orthotropic thermal conductivity – *k*_*z*_ and *k*_*θ*_ – are not measured, since the theoretical derivation shows that the core temperature is independent of these properties.

### Calibration and Validation of IR Camera Measurements

A FLIR A6703 InSb infrared camera is used for transient surface temperature measurements needed for determination of the core temperature as a function of time. Experiments are first carried out to establish the accuracy of infrared temperature measurement, particularly in a transient setting. The accuracy of infrared based temperature measurement is strongly dependent on the quality of calibration. In this case, the same calibration settings established in past experiments are used^15^, and experiments are carried out to track the known temperature of a surface as a function of time with the infrared camera. An Instec HCS662V thermal stage is used for this purpose. The temperature of the stage can be controlled in a wide temperature range. A desired ramp rate can also be specified between two temperatures. A thermocouple embedded within the stage provides the stage temperature as a function of time, to which the infrared temperature measurement can be compared for validation.

The infrared camera is mounted above an optical breadboard, and the stage is placed directly below, as shown in the inset of Supplementary Figure S7. A thin graphite film is sprayed using a DGF aerosol spray on a small region of the thermal stage to increase surface emissivity. Starting at room temperature, the stage is set to reach a temperature of 60 °C with a ramp rate of 5 °C per minute. Temperature of the graphite-coated stage surface is monitored through the embedded thermocouple at 1 Hz frequency. The stage temperature is also measured using the infrared camera during this time at the same rate. Infrared measurements are carried out both without and with the specification of emissivity of the graphite surface, which are then compared with the thermocouple measurement to establish the accuracy of infrared temperature measurement.

### Experimental setup for core temperature measurement

A picture of the experimental setup for measuring the transient core temperature of the test cell using transient surface temperature measurements is shown in Figure 1(a). The thermal test cell is mounted on two thin, foam risers to minimize conduction heat loss, and placed on an optical breadboard directly under the infrared camera. The outer surface of the thermal test cell is coated with the same graphite film used for calibration experiments. The measurement and data acquisition flow is shown schematically in Figure 1(b). Internal heat is generated inside the test cell through resistive heating in the metal foil due to a DC heating current sourced from a GW INSTEK GPD-4303S power supply. The heating current can be switched on and off in order to produce time-varying heat generation rate. Potential difference across the resistive heater is also monitored using a Keithley 2100 digital multimeter controlled by LabView software in order to get measurements of heat generation rate as a function of time. A Fugetek HT-07530D12 computer fan is used in forced convection experiments to provide cooling. The fan is placed at the same height as the axis of the cell to ensure direct and symmetrical impingement of air from the fan on to the test cell. A variable resistor controller is used for fan speed control. Data acquisition from the thermocouple embedded in the core of the test cell is carried out using a NI-9213 DAQ thermocouple module and controlled by LabView software. Infrared temperature measurements as well as thermocouple measurements are taken once every 3 seconds. These measurement rates are appropriate since this system has a much larger time constant than the data acquisition interval.

### Experiments

A number of experiments are carried out in a variety of heating and cooling conditions in order to measure the core temperature of the test cell as a function of time using the method described in the previous sub-section, and to compare with actual core temperature from the embedded thermocouple. In each case, the test cell starts at ambient temperature, and is supplied with a constant or time-varying heating current. The temperature field on the surface of the cell is measured as a function of time using the infrared camera until thermal steady state is reached. In order to determine the heat generation rate, the electrical resistance of the test cell is measured in advance using a small test current that minimizes self-heating. It is also confirmed that the resistance of the test cell does not increase appreciably due to temperature rise during the experiment.

Temperature distribution around the circumference of the test cell at mid-height is extracted from the temperature field data from the infrared camera at a number of time points during each experiment. Since the infrared camera views a projection of the curved surface of the cylinder, a transformation is applied on the data in order to determine the surface temperature distribution as a function of *θ*. The axial dependence of temperature is examined as a function of time in order to rule out significant axial thermal conduction effects. It is found that there is minimal temperature variation in the axial direction, which justifies the assumption of an infinite cylinder in the thermal model.

Three sets of experiments are carried out. First, the core temperature of the cell is measured as a function of time using transient surface temperature measurements at a number of heating powers while the test cell is cooled via natural convection conditions. Subsequently, a set of experiments is carried out at constant heating power with forced convection cooling from a fan operating at various speeds. Finally, experiments are carried out for each of these two cooling conditions, with time-varying Joule heating. In these experiments, heat generation in the cell is switched on and off for periods of 15 minutes each, over a total of three cycles. This mimics periodic heat generation in a Li-ion cell undergoing cyclic charge and discharge^9^.

A stabilization time of five minutes is provided in each experiment with air flow from the fan before the experiment commences. An extended cool-down period is provided between experiments to ensure that each experiment begins at room temperature.

In each experiment, the transient core temperature of the cell is determined using the experimentally measured surface temperature distribution in conjunction with the theoretical model. These core temperature measurements are compared against measurements from the embedded thermocouple in the test cell to validate the non-invasive transient core temperature measurement method.

## Conclusions

In this paper, a novel, non-intrusive method for determining the temperature inside solid bodies has been introduced and validated using experiments on a cylindrical thermal test cell. By utilizing the measured surface temperature distribution as a function of time, this method accurately and non-invasively predicts the core temperature as a function of time. This method, illustrated here for a cylindrical body can be easily extended to bodies of other shapes through analysis similar to one shown in this paper. A non-invasive approach for internal temperature measurement is attractive for a variety of engineering systems where insertion of a temperature sensor inside the body is simply not possible. This capability could be used for smart, thermally-aware performance optimization, as well as for ensuring thermal safety.

## Additional Information

**How to cite this article**: Anthony, D. *et al*. Non-invasive, transient determination of the core temperature of a heat-generating solid body. *Sci. Rep.* **6**, 35886; doi: 10.1038/srep35886 (2016).

**Publisher’s note:** Springer Nature remains neutral with regard to jurisdictional claims in published maps and institutional affiliations.

## Supplementary Material

Supplementary Information
