# Supplementary material for: Non-invasive, transient determination of the core temperature of a heat-generating solid body
Source: Sci Rep. 2016 Nov 2;6:35886. doi: 10.1038/srep35886 (PMC5090205; doi:10.1038/srep35886)
Supplement: Supplementary Information [file srep35886-s1.pdf]

Supplementary Information

Non-invasive, transient determination of the core temperature of a heat-generating solid body

Dean Anthony, Daipayan Sarkar, Ankur Jain*

Mechanical and Aerospace Engineering Department
University of Texas at Arlington, Arlington, TX, USA.

* – Corresponding Author: email: jaina@uta.edu;
500 W First St, Rm 211, Arlington, TX, USA 76019
Ph: +1 (817) 272-9338; Fax: +1 (817) 272 2952

Theoretical Derivation of Equation (1)

This section derives a relationship between the transient core temperature, $T_{core}(t)$ at $r=0$ and the transient surface temperature distribution, $T_0(\theta,t)$ of a heat-generating infinite cylinder. While similar solutions have been derived in the past using the method of Green's functions (for example, Özişik, M.N., Heat conduction, 2nd Ed., John Wiley & Sons, 1993), this derivation takes into account circumferential variation in T_0, as well as anisotropic thermal conduction within the cylinder.

This derivation leads to an expression for $T_{core}(t)$ that forms the basis for the technique to non-intrusively determine the core temperature.

Constant heat generation rate

This sub-section considers an infinite cylinder of radius R generating heat at a uniform and constant rate Q, shown schematically in Supplementary Figure S1. The cylinder is assumed to

have orthotropic thermal conductivities, with values of k_r and k_θ in the radial and circumferential directions respectively. The circumferentially-varying, transient temperature distribution along the outer surface at $r=R$, given by $T_0(\theta,t)$ is assumed to be known, for example, through an infrared measurement. The cylinder is assumed to be at uniform temperature initially.

The transient temperature distribution in the cylinder can be determined by solving the governing energy conservation equation subject to appropriate boundary conditions. Using the separation of variables approach (Özişik, M.N., Heat Conduction, 2nd Ed., John Wiley & Sons, 1980), the temperature field may be split into two parts and assumed to be of the following form:

$$T(r,\theta,t)=T_1(r,t)+T_2(r,\theta,t)=\left[\frac{Q\left(R^2-r^2\right)}{4k_r}+\sum_{m=0}^{\infty}A_n J_0\left(\lambda_{0n}r\right)e^{-\alpha_r\lambda_{n0}^2\cdot t}\right]+\sum_{m=0}^{\infty}\sum_{n=1}^{\infty}B_{mn}(t)J_{m\sqrt{\frac{k_\theta}{k_r}}}\left(\lambda_{mn}r\right)\cos(m\theta) \quad \text{(S.1)}$$

where T_1, comprising the first two terms, accounts for internal heat generation, and T_2, comprising the third term accounts for time- and θ-dependent temperature at $r=R$.

Here, J refers to the Bessel function of the first kind, and the radial norm $N_{r,n}$ is given by

$$N_{r,n}=\frac{R^2 J_1(\lambda_{0n}R)^2}{2} \quad \text{(S.2)}$$

and the eigenvalues λ_{mn} are obtained from the roots of $J_{m\sqrt{\frac{k_\theta}{k_r}}}$. Specifically, λ_{0n} are obtained from the roots of J_0. Using the orthogonality principle (Özişik, M.N., Heat Conduction, 2nd Ed., John Wiley & Sons, 1980), A_n are given by

$$A_n=\frac{-\frac{Q}{4k_r}\int_0^R\left(R^2-r^2\right)\cdot rJ_0\left(\lambda_{0n}r\right)dr}{N_{r,n}} \quad \text{(S.3)}$$

The coefficient functions $B_{mn}(t)$ must be determined such that the solution satisfies the governing equation and boundary conditions.

Note that the core temperature $T_{core}(t)$ may be expressed in terms of the unknown coefficient functions $B_{nm}(t)$ by putting $r=0$ in equation (S.1)

$$T_{core}(t) = T_{1,core}(t) + T_{2,core}(t) = \frac{QR^2}{4k_r} + \sum_{n=1}^{\infty} A_n e^{-\alpha_r \lambda_{n0}^2 \cdot t} + \sum_{n=1}^{\infty} B_{0n}(t) \tag{S.4}$$

Equation (S.4) shows that even though all coefficient functions $B_{mn}(t)$ are needed to determine the general temperature distribution $T(r,\theta,t)$, only the coefficient functions $B_{0n}(t)$ are needed for measuring the core temperature. To do so, the expression for $T_2(r,\theta,t)$ from equation (S.1) is integrated with respect to θ to eliminate all $B_{mn}(t)$ for m>0, followed by use of the method of undetermined parameters (Myers, G.E., Analytical Methods in Conduction Heat Transfer, 2nd Ed., AMCHT Publications, 1998) to derive the following ordinary differential equation for $B_{0n}(t)$

$$\frac{dB_{0n}}{dt} = -\alpha_r \lambda_{0n}^2 B_{0n} + \frac{\alpha_r \lambda_{0n} R J_1(\lambda_{0n} R)}{2\pi N_{r,n}} \int_0^{2\pi} T_0(\theta,t) d\theta \tag{S.5}$$

Further, based on the initial condition for the temperature field, the following initial condition applies for $B_{0n}(t)$

$$B_{0n}(0) = 0 \tag{S.6}$$

A solution for equation (S.5) subject to (S.6) is given by

$$B_{0n}(t) = \frac{\alpha_r \lambda_{0n} R J_1(\lambda_{0n} R)}{N_{r,n}} \int_0^t w_{0I}(\tau) \exp\left[-\alpha_r \lambda_{0n}{}^2 (t-\tau)\right] d\tau \tag{S.7}$$

where $w_{0I}(\tau)$ is the circumferentially averaged value of the measured temperature at the outside surface, given by

$$w_{0I}(\tau) = \frac{1}{2\pi}\int_0^{2\pi} T_0(\theta,\tau)\,d\theta \qquad \text{(S.8)}$$

This completes the determination of the core temperature $T_{core}(t)$, which is given by equation (S.4), where the coefficient functions are given by equations (S.2) and (S.7).

Effect of time-varying heat generation rate

The previous sub-section assumed a time-invariant internal heat generation rate in the cylinder. In this sub-section, a more general case is considered where the heat generation varies with time, given by $Q(t)$. Time-varying heat generation is encountered in several engineering applications, where the measurement of core temperature in response to $Q(t)$ is of interest.

To develop a technique for internal temperature measurement for this case, the governing energy equation needs to be revisited. As shown in equation (S.1), the components T_1 and T_2 of the overall temperature distribution account for temperature rise due to internal heat generation and due to the time-dependent surface temperature respectively. As a result, variation in Q with time affects only the T_1 component of the temperature field. A Laplace transform approach is used to derive a solution for $T_1(r,t)$ for when Q varies with time. Taking Laplace transform of the governing equation and boundary condition for T_1 results in the following:

$$\left(\frac{d^2\overline{T}_1}{dr^2} + \frac{1}{r}\frac{d\overline{T}_1}{dr}\right) - \frac{s}{\alpha_r}\overline{T}_1 + \frac{\overline{Q}(s)}{k_r} = 0 \qquad \text{(S.9)}$$

Where $\overline{Q}(s)$ is the Laplace transform of the heat generation rate *Q(t)*. $\overline{T}_1$ also satisfies

$$\frac{d\overline{T}_1}{dr} = 0 \qquad \text{at } r=0 \qquad \text{(S.10)}$$

and

$$\overline{T}_1 = 0 \qquad \text{at } r=\text{R} \qquad \text{(S.11)}$$

The solution to this ordinary differential equation can be shown to be given by

$$\overline{T}_1 = \frac{\alpha_r \overline{Q}}{sk_r}\left(1 - \frac{I_0\left(\sqrt{\frac{s}{\alpha_r}}r\right)}{I_0\left(\sqrt{\frac{s}{\alpha_r}}R\right)}\right) \qquad \text{(S.12)}$$

where I_0 is the modified Bessel function of the first kind.

As a result, the core temperature $T_{core}(t)$ for an infinite cylinder can be written as

$$T_{core}(t) = T_{1,core}(t) + T_{2,core}(t) = L^{-1}\left[\frac{\alpha_r \overline{Q}}{sk_r}\left(1 - \frac{1}{I_0\left(\sqrt{\frac{s}{\alpha_r}}R\right)}\right)\right] + \sum_{n=1}^{\infty} B_{0n}(t) \qquad \text{(S.13)}$$

where L^{-1} refers to the inverse Laplace transform. Note that $B_{0n}(t)$ is given by equation (S.7).

For a given *Q(t)*, the inverse Laplace transform may be determined analytically when possible, or numerically using inverse Laplace transform numerical algorithms. This provides a technique to predict the core temperature of the cylinder in presence of a time-varying heat generation within the body.

Supplementary Figures

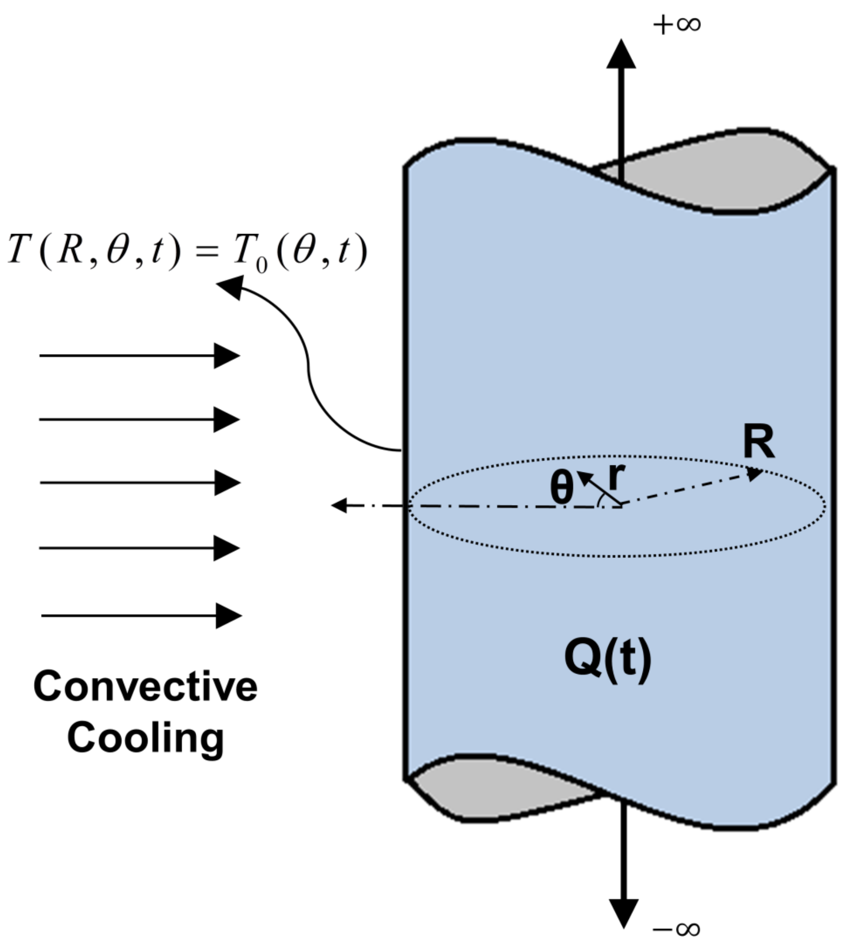

Supplementary Figure S1: Schematic of the geometry of a heat-generating infinite cylinder. The goal of the measurement is to determine the core temperature $T(r=0,t)$ as a function of time from measured surface temperature $T_0(\theta,t)$ as a function of time.

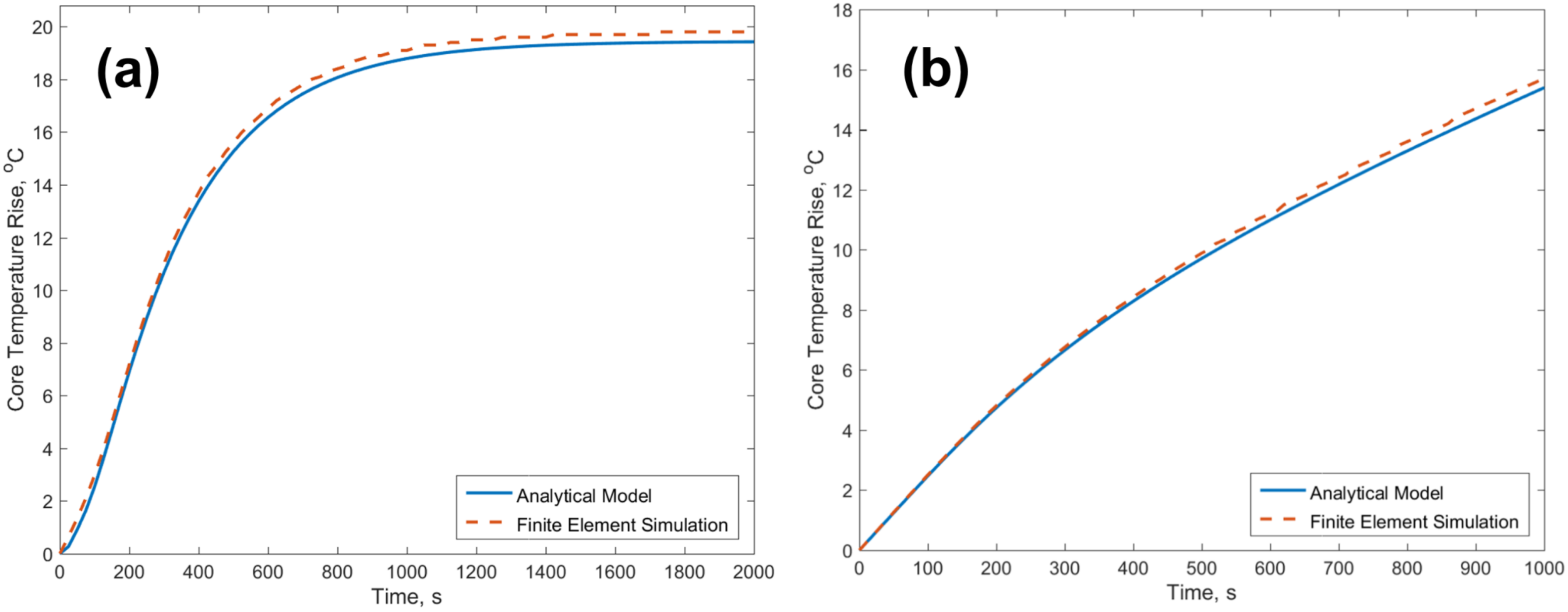

Supplementary Figure S2: Comparison of transient core temperature predicted by the theoretical model with finite-element simulation results for two surface temperature conditions, (a) constant T_0=10°C, (b) linearly increasing $T_0(t) = 10 \times \frac{t}{1000(s)}$ °C. Both model and finite-element simulations assume a 13 mm radius cylinder with 57,954 W/m^3 heating, and the same thermal properties as the experimental thermal test cell.

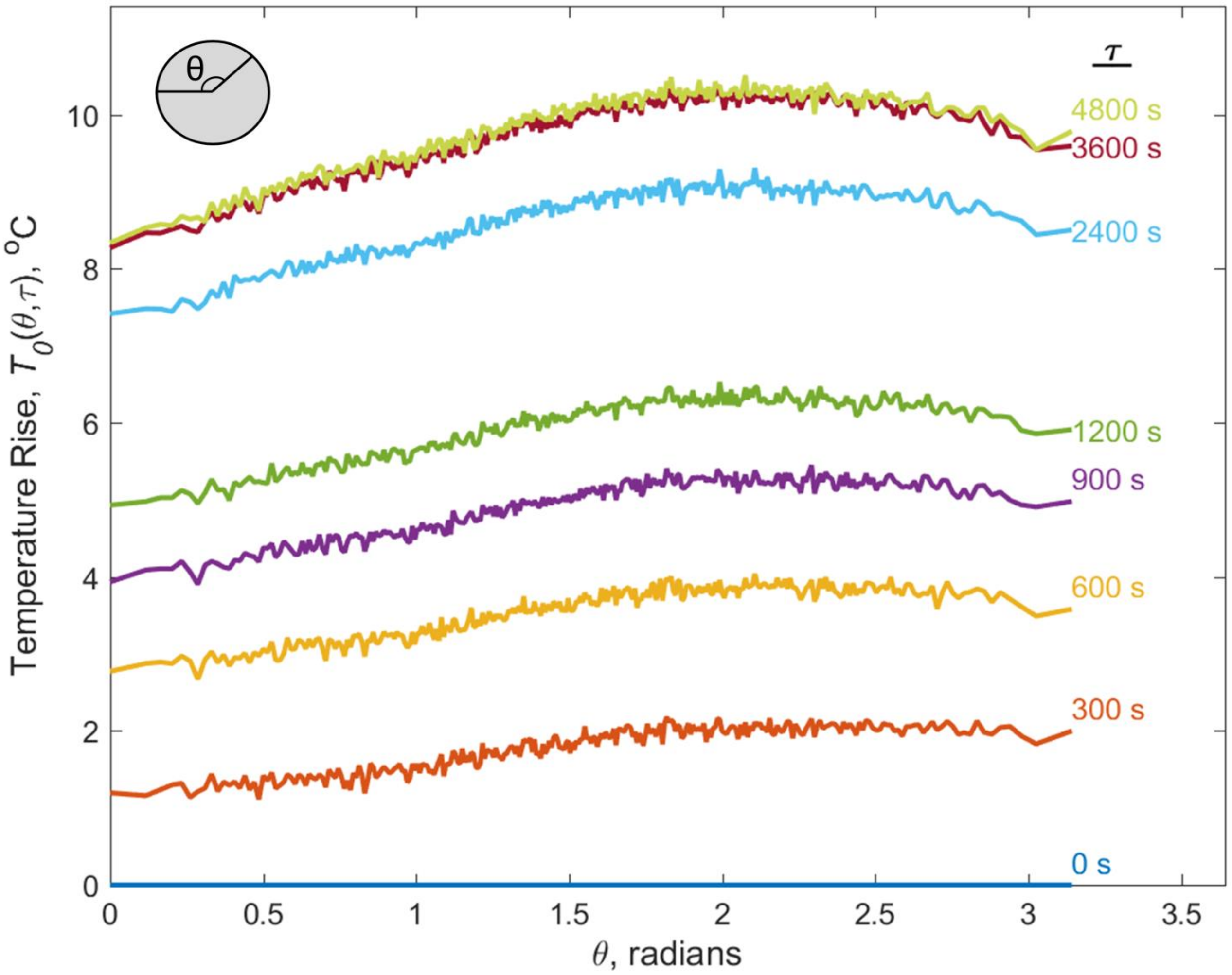

Supplementary Figure S3: Measured surface temperature distribution around the cylinder $T_0(\theta,\tau)$ at a number of times following the start of heating for 0.86 W heating power in the thermal test cell in free convection cooling conditions.

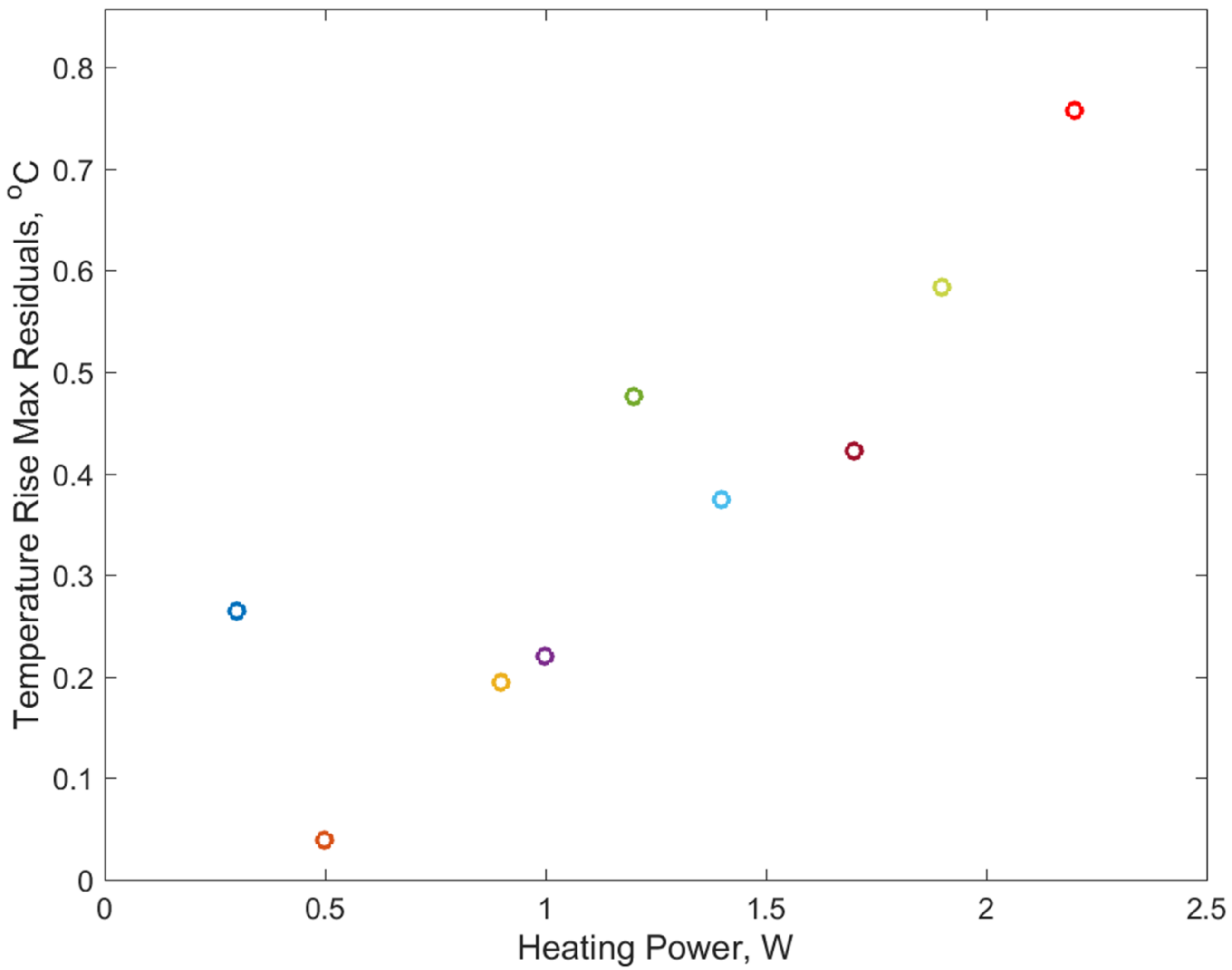

Supplementary Figure S4: Plot of the maximum residual over the entire experiment duration as a function of heating power for Figure 3.

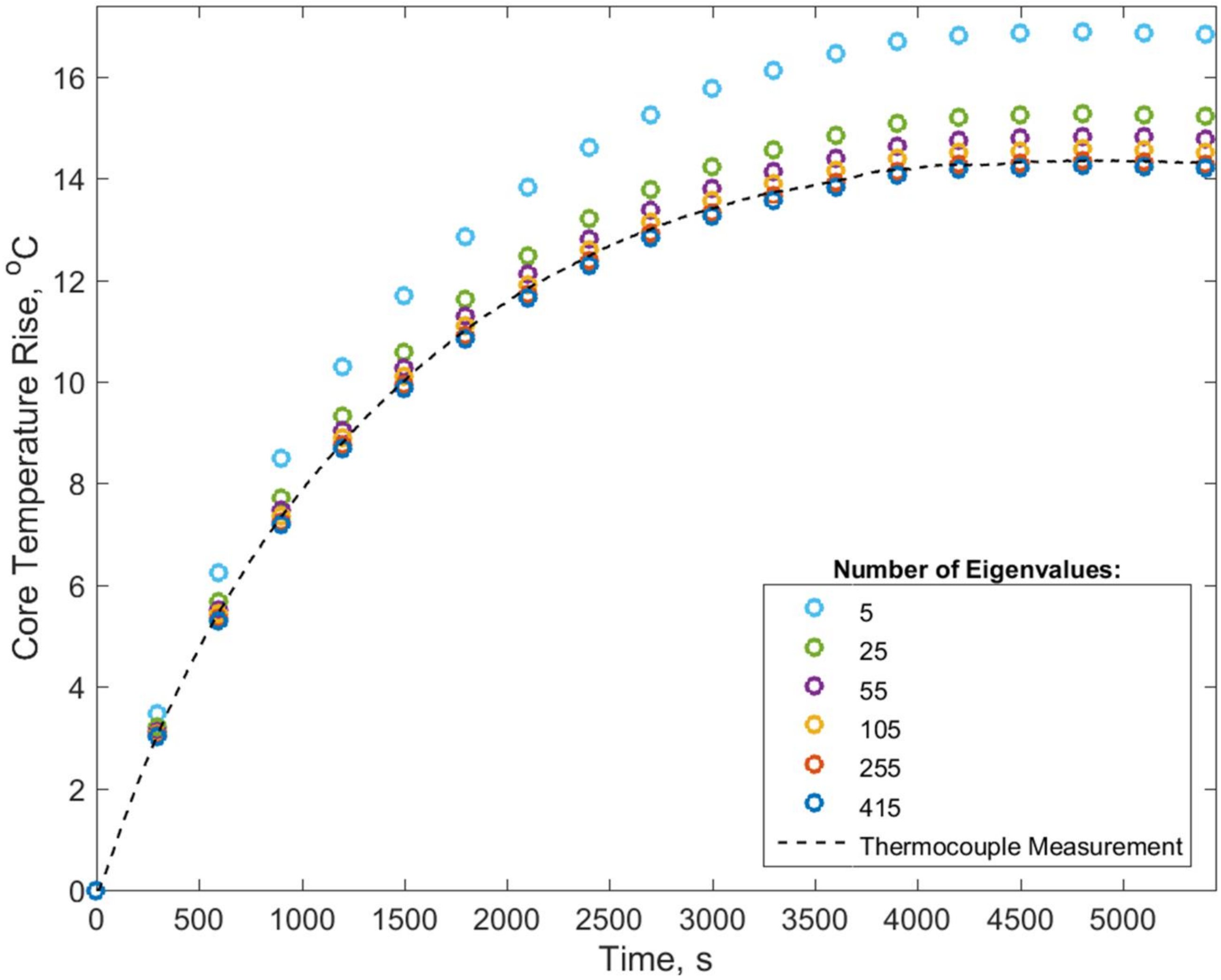

Supplementary Figure S5: Predicted variation of core temperature as a function of time for different number of eigenvalues considered for the $B_{0n}(t)$ term in equation (1). For comparison, the embedded thermocouple measurement is also shown as a broken line.

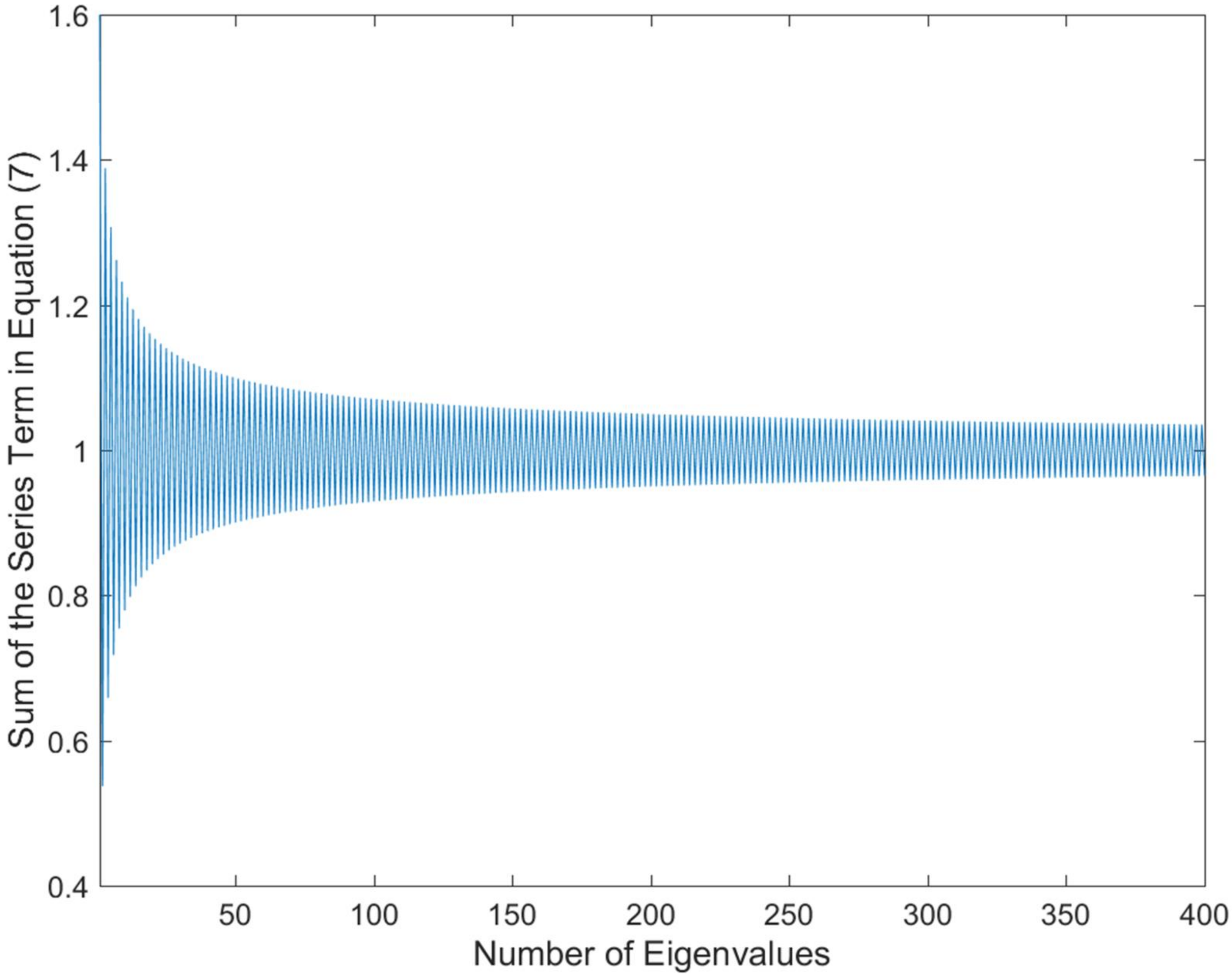

Supplementary Figure S6: Convergence of the infinite series in equation (7) with respect to the number of eigenvalues considered, showing slow series convergence.

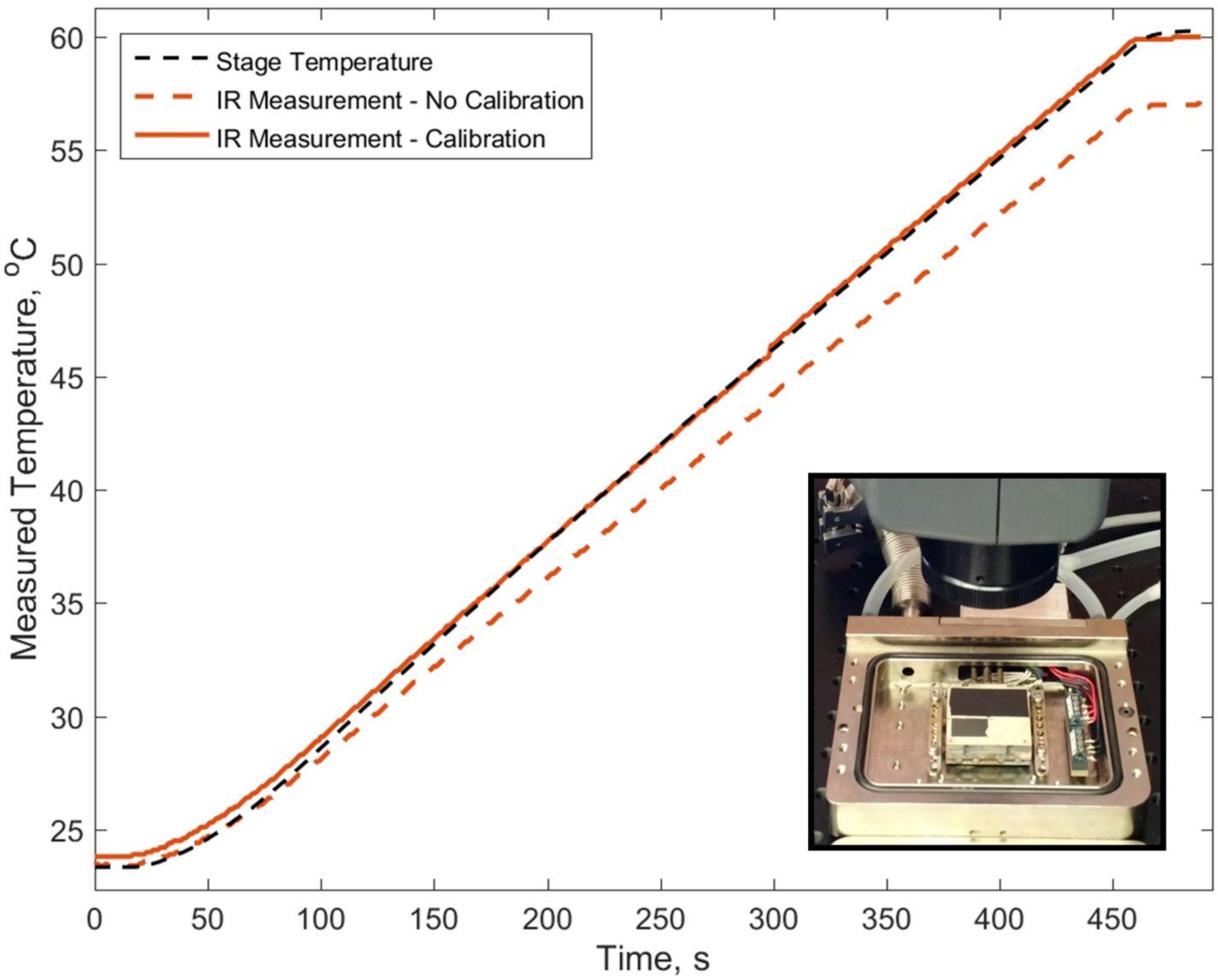

Supplementary Figure S7: Comparison of IR camera measured temperature of a surface with a known surface temperature as a function of time, during a temperature ramp from room temperature to 60 °C at a rate of 5 °C/min. IR data are shown both with and without calibration in red lines. The known surface temperature is also plotted in black for comparison.
